# Mitochondrial network morphology: building an integrative, geometrical view

**DOI:** 10.1186/1741-7007-11-71

**Published:** 2013-06-24

**Authors:** Susanne M Rafelski

**Affiliations:** 1 Department of Developmental and Cell Biology and Center for Complex Biological Systems, University of California, Irvine, CA 92697, USA

## Abstract

The morphology of mitochondrial networks is complex and highly varied, yet vital to cell function. The first step toward an integrative understanding of how mitochondrial morphology is generated and regulated is to define the interdependent geometrical features and their dynamics that together generate the morphology of a mitochondrial network within a cell. Distinct aspects of the size, shape, position, and dynamics of mitochondrial networks are described and examples of how these features depend on one another discussed.

## 

It remains an intriguing mystery how a cell generates and regulates the morphology of its organelles. Consider the mitochondria. They are the powerhouse of the cell, centers of respiratory ATP production and key organelles in essential lipid metabolism pathways. Within a cell, mitochondria appear in a range of forms from numerous small individual organelles, as most often depicted in textbook illustrations, to a single large interconnected membrane-bound tubular network, depending on environmental conditions, cell type, and organism (Figure 1) [1-4]. What are the underlying principals and rules that shape an organelle as geometrically complex as the mitochondrion? What aspects of mitochondrial morphology arise from dynamic self-organizing principles, as seen for other intracellular structures [5], and what aspects must be actively controlled to respond to the functional needs of the cell.

**Figure 1.**
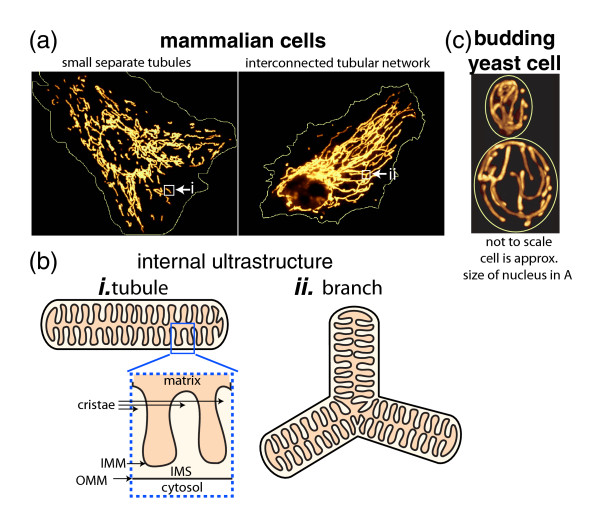
**Examples of mitochondrial networks at the micron and nanometer scales.** (**a**) Mitochondrial networks can vary from separated structures to interconnected networks. Indian Muntjac deer skin fibroblast (left) and BPAE (bovine pulmonary artery endothelial) cell (right), both expressing a pEYFP-mitochondrial plasmid vector to label mitochondria (yellow-orange). The thin yellow line is an approximate outline of the cell. White arrows point to small boxes indicating either a tubule (left) or tubule branch (right) that are illustrated in (b). (**b**) Diagram of the organization of the mitochondrial membranes (ultrastructure) at the nanoscale for (i) a tubule and (ii) a tubule branch. Abbreviations in (i) include the outer and inner mitochondrial membrane (OMM and IMM, respectively) and the intermembrane space (IMS). (**c**) The mitochondrial network in a budding yeast cell. Thin yellow line indicates the outline of the mother (larger, bottom) and bud (smaller, top) compartments. Image is a maximum intensity projection of a three-dimensional z-stack. The size of the cell is approximately the same size as the nuclei in the mammalian cells shown in (a). Images in (a) are reproduced and slightly altered courtesy of and with permission from Michael Davidson (Florida State University) and are featured on the Nikon MicroscopyU website [57].

To answer these questions, we need a multi-scale view of how the morphology of a mitochondrial network in the cell is achieved. We must consider both a) how molecular interactions at the nanometer scale generate the tubular structure of mitochondria and b) how these tubules are then organized into the functional, dynamic mitochondrial networks observed in cells at the intermediate micron scale. Traditional approaches have successfully identified molecular alterations that generate observable aberrant mitochondrial morphology (see ‘Shape’ and ‘Dynamics’ sections below). However, in most cases, descriptions of these phenotypes are qualitative. Measurements of the severity and penetrance of the phenotype are generally limited to classifications into arbitrary categories. These categories do successfully distinguish the specific morphological classes in a given study, but then require re-classification when additional subtle features of morphology are identified that were not accounted for in previous work. Instead, we need to develop new methods to obtain systematic quantitative descriptors of morphology so that we can rigorously and universally measure normal and aberrant mitochondrial morphology. This is especially important because building an integrative view of mitochondrial networks will require multi-scale computational modeling, which necessitates quantitative experimental data for comparing model to experiment and testing model predictions.

There are several geometric features that together define the overall morphology of mitochondrial networks. These include the size, shape, and position of mitochondria as well as the dynamics of how these features change with time (Figure 2). These geometrical features can be considered both at the nanometer scale, referring to the morphology of the individual mitochondrial tubules, as well as at the micron scale, referring to the overall organization of the entire mitochondrial network within a cell. While these features can be defined distinctly, they do not exist independently; rather, they all inform and affect each other. In this article I will use mitochondrial networks in the budding yeast *Saccharomyces cerevisiae* (Figure 1c) to define and illustrate distinct aspects of each of these geometrical features of mitochondrial morphology at the scale both of mitochondrial tubules and of the mitochondrial network as a whole. I will also discuss the interdependence between these features and their relation to mitochondrial function. Finally, I will discuss some important future directions needed for an integrative understanding of mitochondrial morphology.

**Figure 2.**
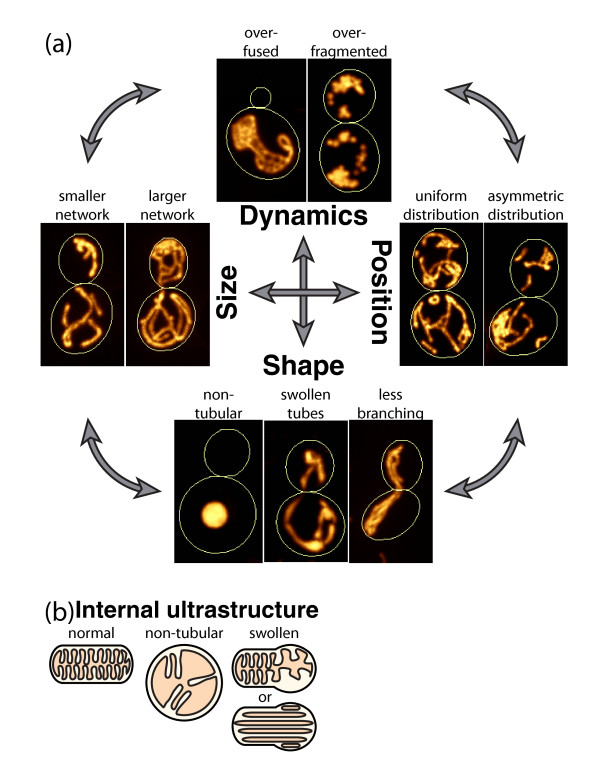
**Examples of the geometrical features and their dynamics that together generate the overall morphology of mitochondrial networks.** Mitochondrial networks in budding yeast are used as a model system to highlight these features but the features are all equally applicable to other organisms and cell types. Mitochondria are labeled by a matrix-targeted fluorescent protein [11] and cell boundaries are shown with thin yellow lines as in Figure 1c. The specific condition or mutation generating each example image is not specified because a variety of mutations can alter each of these geometric features and the purpose of the figure is to illustrate the features themselves in a more general way. Size: example cells with a larger and a smaller mitochondrial network arising from growth in respiratory and non-respiratory conditions, respectively. Shape: example cells with mitochondria lacking their normal underlying tubular structure or exhibiting swollen irregular tubules due to changes in internal membrane organization are shown. Mitochondria in the third cell display normal tubular structure but instead the network contains many fewer branch points than normal. These tubules lie parallel to each other without connections between them. This network is also less uniformly distributed within the cell, highlighting the interdependence between features (depicted by the large gray arrows). Position: example cells with a more uniform versus more asymmetric distribution within the cell. Note the large area of the cell devoid of any mitochondria in the latter case. Dynamics: example cells displaying the resultant over-fragmented and over-fused mitochondrial networks generated when either fusion or fission dynamics are reduced. The final topology of these networks is also greatly altered as a result, once again emphasizing the interdependence of these features. As described in the text, the topology itself may affect the local fission and fusion dynamics as well as the ability of sub-regions of the network to move around within the cell. This may explain the less uniform distribution of the over-fused networks and re-emphasizes the importance of considering both directions of interdependence between features.

## Size

The most fundamental geometrical feature of any organelle is its size. In the case of mitochondria, size can be measured in several different ways. Metrics of mitochondrial size include the width of the mitochondrial tubules and their overall length within the cell, which together determine the total volume of mitochondria bounded by their outer membrane. In addition to the volume, the surface area of all of the tubules making up the mitochondrial network may also be considered. However, due to the tubular geometry, the surface area and volume of the mitochondrial network will both be a function of length and thus all three metrics will differ merely by a multiplication factor derived from the tubule radius. The only deviation from this proportionality would come from the relatively small branching regions of the network.

In addition to the physical size of the organelle bounded by the outer membrane, one must consider mitochondrial size in terms of the ‘amount of mitochondria’ in a cell, which can also be measured using a biochemical approach. It is well known that the internal membrane organization and protein composition of mitochondria can dramatically change in response to changes in functional state [6]. This could lead to a change in the ratio of protein to lipid content or changes in protein mass that are much greater than a change in the physical bounding volume of the organelle. For example, an up to 10- to 20-fold increase in transcription and expression levels of mitochondrial proteins can be seen in respiring versus non-respiring yeast cells [7,8], which is much greater than the 3-fold change in mitochondrial volume in these conditions (Figure 2a) [9]. Another important size-related issue to consider is the difference in the absolute size of mitochondria in a cell versus the ratio of mitochondrial size to cell size, the ‘mitochondrial volume ratio’, which is effectively a measure of the spatial density or ‘concentration’ of mitochondria within a cell [10,11]. All of these aspects of size need to be considered when describing how the geometrical feature of size contributes to overall mitochondrial morphology or function.

The size of the mitochondrial network at any given moment arises from the combination of mitochondrial biogenesis (creation of new mitochondrial material) and mitophagy (mitochondrial autophagy, which degrades mitochondria). These processes can respond to the needs of the cell. The increase in both the mitochondrial protein content and the physical size of the mitochondrial network when yeast cells transition from non-respiratory to respiratory conditions is an example of the upregulation of biogenesis to generate increased mitochondrial content. On the other hand, mitophagy is induced when cells experience a variety of stresses [12]. For example, growing yeast cells in nitrogen-depleted media induces both general autophagy and mitophagy to generate nitrogen for essential cellular processes. Biogenesis and mitophagy have to be regulated to maintain the proper mitochondrial content during normal cell growth.

## Shape

Definitions of mitochondrial shape depend heavily on what spatial scale is considered. At the nanometer scale lipids and proteins assemble into distinct mitochondrial membranes and compartments to generate the internal ultrastructure of mitochondrial tubules (Figure 1b). Yet at the micron scale, mitochondria also exhibit a shape, the organization of the entire mitochondrial network as a whole within the cell (Figure 1a,c). The basic mitochondrial unit has a tubular form and generally exhibits a constant diameter throughout the cell. Mitochondria are shaped into tubules through their internal membrane organization, including cristae architecture, proper association between the different membranes, segregation of proteins into distinct membrane domains, and interactions with other organelles. Manifestations of alterations or defects in this mitochondrial ultrastructure include swollen tubules of inconsistent diameter and giant spherical mitochondria exhibiting disorganized internal membrane structures (Figure 2) [13-15]. The molecular mechanisms permitting mitochondrial tubulation are still not understood although they are under active investigation [16,17]. Recently, several groups identified a mitochondrial protein complex vital to internal membrane organization that seems to be central in coordinating mitochondrial ultrastructure, biogenesis, mitochondrial DNA (mtDNA), and perhaps even mitochondrial-endoplasmic reticulum (ER) membrane connections [18-21]. The complex was named MitOS (mitochondrial organizing structure) [19], MICOS (mitochondrial contact site) [18], or MINOS (mitochondrial inner membrane organizing system) [20] and includes the proteins Fcj1, Aim5, Aim13, Aim37, Mio10, and Mio27 (reviewed in [22]). It is localized to the inner membrane and intermembrane space, is constrained to the regions of the inner membrane that interact with the outer membrane, and is excluded from the cristae. The complex is believed to function in separating the inner membrane into distinct spatial domains and thus contributing to the formation of the cristae. Mutants in these proteins dramatically alter internal membrane organization, abolishing the normal organization of the cristae and instead generating very long, stacked membranes (Figure 2b).

In the absence of any gross defects in internal organization, mitochondria maintain their underlying tubular shape and diameter. These tubules are further organized into a network structure spanning the cell at the micron scale. This network also has a shape, with the connectivity of the network and its distribution within the cell varying within different regions of the cell and from cell to cell. The mitochondrial network can exist at the extremes as a collection of small separate sub-networks or one single, interconnected organelle (see dynamics section below). Mitochondrial networks can also exhibit more subtle variations in their topology - for example, containing different numbers of tubule branchpoints (Figure 2a). This ‘topological’ aspect of mitochondrial shape can be quantified by considering mitochondrial networks as mathematical graphs with edges (tubules) and nodes (branchpoints connecting tubules). The pure topology of the network can then be measured [23] by considering how many edges are connected at each node. By further assigning a physical location within the cell to each node and along the length of each edge [11], the network can also be considered a ‘geometric graph’, permitting additional analyses that incorporate the spatial component of the networks to describe the shape of mitochondria at the scale of the entire cell. For example, a more over-connected network would exhibit a greater ‘average degree’ (the number of edges that enter each node, averaged over the entire network) while a network with more long tubules between branchpoints might exhibit a greater average length per edge (average of the lengths of all the edges in the network). Applications of standard network analysis methods will be a useful tool to quantify the micron-scale network morphology of mitochondria.

## Dynamics

Formally, the term ‘mitochondrial dynamics’ includes all of the different ways that the geometrical features of mitochondrial networks change over time. More commonly, ‘mitochondrial dynamics’ refers to the fission and fusion dynamics that constantly remodel the mitochondrial network. Fission cuts a tubule into two while fusion can link two tubules together to form a longer tubule or a branch. The balance of fission and fusion is important for shaping mitochondrial networks; more fission than fusion leads to over-fragmented networks while more fusion than fission leads to over-connected networks compared to normal, wild-type networks (original papers reviewed in [24]) (Figure 2a). If fusion is increased sufficiently over fission, all the separated mitochondrial regions have the potential to be interconnected into a single mitochondrial network. The canonical ‘individual’ mitochondrion or small mitochondrial sub-networks arise due to fission events. However, these are in fact not separate individuals but transiently separated sub-regions of the entire mitochondrial network that is in some state of fission and fusion at any given time. Photoconversion studies of matrix-targeted reporter proteins in yeast have shown that the entire mitochondrial network is completely interconnected, and thus the matrix contents of the entire network mixed, within 5 seconds after initiating the photoconversion in half of cells while in the other half, 30 to 95% of the network is interconnected within that time. By 10 minutes the entire network of all cells has become interconnected and all its matrix contents mixed [2]. Since the average rate of fission and fusion in yeast cells is about one per minute per cell [25], this suggests that in most cells most of the network is already interconnected and in the rest of cells it becomes so through several fusion events. In mammalian cells, rates of fission and fusion are slower [26] and mitochondrial networks are also much larger. A decreased rate of dynamics and increased size together should lead to a longer timeframe over which mitochondrial networks are interconnected, consistent with experimental observations [27,28]. Further, because fusion requires a mitochondrial membrane potential [29,30], sub-regions of the network that have lost the capability to function properly and generate this membrane potential can remain separated indefinitely, and are believed to be targeted for mitophagy [28].

The molecular machines responsible for exerting the fission and fusion dynamics have been extensively studied. Fission occurs through the Dnm1p protein, which is a DRP (dynamin-related protein) GTPase that can assemble into a spiral structure and constrict upon activation by accessory proteins Mdv1p and Fis1p [31,32]. Intriguingly, the ER is associated with sites of slight mitochondrial tubule constrictions that become future fission sites. These constrictions may enable Dnm1p binding and more efficient fission events [33]. Recently, activation of actin polymerization activity via formins has also been implicated at these ER-associated sites of mitochondrial fission, suggesting that a possible mechanism for the ER-associated mitochondrial constriction may be through regulation of cytoskeletal dynamics [34]. Fusion involves two other DRPs, Fzo1p and Mgm1p, for outer membrane and inner membrane fusion, respectively, with the two processes being coordinated by Ugo1p [35]. The fusion process requires GTP binding and hydrolysis as well as a membrane potential [29,30].

**Figure 3.**
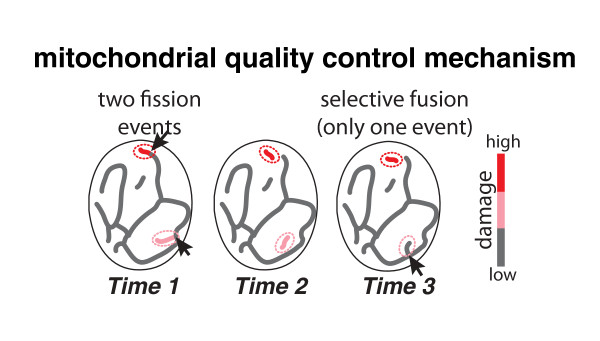
**Diagram of the ‘mitochondrial quality control mechanism’ shows three sequential timepoints of fission and fusion.** Arrows in Time 1 represent two fission events. Arrow in Time 3 represents the re-fusion of the pink fragment to the network and dissipation of its damage. The red fragment has accumulated too much damage and cannot re-fuse with the network. It will be targeted for mitophagy. Color bar represents the amount of mitochondrial damage.

In addition to regulating the topology of the mitochondrial network, fission and fusion dynamics may contribute to the homeostatic health of the mitochondrial network. Recently, a ‘mitochondrial quality control mechanism’ has been proposed based on both experimental and computational results [36]. As illustrated in Figure 3, fusion events allow mixing of oxidatively damaged mitochondrial contents generated as a byproduct of respiration. Thus, a damaged mitochondrial fragment can be restored when it re-fuses to the network. Fusion itself is dependent on mitochondrial membrane potential. Therefore, the more damaged a network fragment is, the less likely it is to re-fuse. Instead, if it remains isolated, it is targeted for turnover by mitophagy. Fission, on the other hand, is the mechanism that allows the continuous creation of small mitochondrial fragments, which can either re-fuse with the network or be segregated out if they have become excessively damaged. This model sets up an important framework for deciphering the causality between mitochondrial structure and function.

## Position

The position of mitochondria in the cell at any given time is due to the attachment and movement of mitochondria along dynamic cytoskeletal tracks, the change in position of the tracks themselves, the interactions of mitochondria with other organelles such as the ER and plasma membrane, and the interactions of mitochondrial tubules with each other [33,37-40]. For example, while a cytoskeletal track may direct two tubules to fuse and form a new network branch, once they are fused, the position of the underlying track can change and the mitochondria themselves would still maintain their branched morphology until they undergo further fission or fusion dynamics. The resultant distribution of the mitochondrial network in the cell can range from being uniformly spread out throughout the entire cell, allowing for equal exposure to all areas of the cell, or confined to particular sub-regions of the cell (see T cells as an example [41,42]) (Figure 2a). This distribution directly affects the subcellular regions that have access to higher concentrations of mitochondrial-derived metabolic molecules.

In budding yeast, mitochondria are localized to the cortical periphery of the cell, in part through their interactions with cortical actin cables. A recent study has shown that the cortical localization of mitochondria is also due to interactions with both the plasma membrane and the cortical ER via the Num1p protein [40]. Beyond their localization to the cortex, mitochondria in budding yeast must also be appropriately positioned and redistributed between mother and daughter bud compartments to ensure proper mitochondrial inheritance at cell division. Recently the Ypt11p protein has been implicated in anchoring mitochondria in the bud tip due to its interactions with both the ER and mitochondria [43], although these results are still being debated [44]. Unexpectedly, the inheritance of mitochondrial content at division is asymmetric and this asymmetry can arise by both active and stochastic means [11,45].

## Building an integrative view of mitochondrial morphology and function

One of the most significant challenges to an integrative understanding of mitochondrial morphology is linking mechanisms regulating the ultrastructure at the nanoscale with the resultant network structure at the micron scale. The effect of severe alterations to internal mitochondrial architecture on overall mitochondrial shape is obvious even by fluorescence microscopy using labeled mitochondrial matrix or membrane probes. These deformations can manifest as large spherical-looking mitochondria or mitochondrial networks with regions that look thick, swollen and misshapen (Figure 2). However, visualizing the internal ultrastructure leading to these phenotypes still requires electron microscopy of fixed samples. New super-resolution microscopic techniques have begun to visualize internal mitochondrial membranes [46,47] but are still incapable of rapid three-dimensional timelapse visualizations in live cells. As these new microscopic techniques are improved, they will open up the exciting possibility of visualizing the dynamics of internal mitochondrial membrane reorganization. This will be vital for understanding how the various molecular players interact and contribute to mitochondrial ultrastructure. These methods should also eventually permit simultaneous dynamic imaging of both mitochondrial ultrastructure and network-scale structure, which will facilitate experiments tying together these two very different spatial scales of mitochondrial shape.

A second serious challenge to an integrative view of mitochondrial morphology is the interdependence between its geometrical features. It is immediately apparent, even just in defining mitochondrial size, shape, position, and dynamics, that these four different features of mitochondrial morphology are not cleanly separable from one another. A cell with a larger mitochondrial network will exhibit different topological properties than one with a smaller network. For example, respiring yeast cells, with three-fold larger networks, also exhibit four-fold more branch points [9]. This could arise from the increased volume ratio of tubules within the respiring cells and thus individual tubules having a greater chance of finding each other in space for fusion to occur to generate more branch points. The size of the network could feed back on the frequency of fission and fusion, as has been suggested by the observation that conditions generating larger networks also seem to favor more frequent fission/fusion events [48]. On the other hand, fission and fusion dynamics and network size could impact the position of mitochondria within the cell since dragging a large, interconnected sub-network without the ability to remodel its connections may be much more difficult than moving an unconnected tubular fragment from one part of the cell to another.

Obviously, mitochondrial fission and fusion dynamics will together greatly contribute to mitochondrial network topology, but surprisingly this is not an absolute. Mutants in budding yeast in which all fission and fusion dynamics are seemingly abolished can still generate and maintain an interconnected network, very similar to that of wild-type cells [49]. It is thus not completely understood how and to what extent fission and fusion dynamics contribute to the final topology. Importantly, the organization of the network itself may in turn influence fusion dynamics. Fusion is absolutely dependent on two tubules finding each other in space and time and the type of fusion event further depends on the type of physical interaction between the tubules [50,51]. Fission, on the other hand, generates two new tubule ends that did not exist prior to the fission event and that now each have the ability to move around and potentially generate a new fusion event. The cell may exert additional regulation over how quickly a recently created tubule end is capable of re-fusing to the network [50,51]. However, the fission event itself may contribute to the subsequent fusion dynamics nearby, generating the potential for sub-regions of the mitochondrial network with increased fusion rates. To complicate matters further, fission and fusion proteins may also be co-regulated at the transcriptional level [52], and may interact with each other to modulate their activity [53].

## Computational modeling of mitochondrial networks

Understanding the interdependence between the geometrical features of mitochondrial networks will require a combination of quantitative experimentation and computational modeling. From the computational side, models of mitochondrial network organization can be generated based on any given set of assumptions. The key requirement for these sorts of computational models to be useful is the ability to compare them to ‘real’ networks. Models need to be based on experimentally perturbable parameters with output properties that can be experimentally measured. Several studies have begun carefully documenting the types of fission and fusion events and the biophysical requirements for them to occur [50,51]. These studies are a crucial first step and provide the first extensive quantitative data for incorporation into any computational models of mitochondrial dynamics. Their main limitation is that they focus on specific sub-regions of the mitochondrial network and thus do not encompass the entire network and the way the distribution of fission and fusion impact its organization on the scale of the entire cell. To do so, new methods will be needed that can automatically track all fission and fusion events within a network to provide data on the frequency of remodeling events and their location within the network. Simultaneous measurements of the other network morphology features (size, position, topology) will then permit an analysis of how fission and fusion affect each other and how they are affected by the geometry of the network itself.

Several computational models of mitochondrial networks have been generated. On one hand there exist models that attempt to tie together fission and fusion dynamics with their effect on maintaining mitochondrial health, testing the ‘mitochondrial quality control mechanism’ [54,55] (Figure 3). These models currently only take into account the fission and fusion dynamics themselves, not the physical size, shape and distribution of the mitochondria within the cell. Recently, a new model has been built that for the first time incorporates the fact that mitochondria move in space, can form networks, and undergo continuous biogenesis that is not necessarily coupled to mitochondrial turnover [56]. This model makes testable predictions about where in parameter space real mitochondrial networks should fall to maintain mitochondrial function most successfully. On the other hand, models can be built to determine the underlying requirements for generating the organization of mitochondrial networks without considerations of their functional impact. An example is a recent graph-based model that was built based on a set of simple rules of network connectivity [23]. This model predicts several topological properties that mitochondrial networks should display, and thus can now be tested with directed experiments measuring network topology. A key limitation of this model is that it is purely topological and does not take any spatial information into account. The next generation of computational models will depend on more extensive quantitative experimental measurements of the interdependent geometric features of mitochondrial network morphology and, eventually, simultaneous mitochondrial functional state.
